# Toxic Anterior Segment Syndrome Following Implantable Collamer Lens Implantation in a Patient With Keratoconus and Nystagmus: A Case Report

**DOI:** 10.7759/cureus.96416

**Published:** 2025-11-09

**Authors:** Abeer K Alharbi, Maydaa Dabi, Alaa Almuzaini, Ali Almalki, Hussain Al-Habboubi

**Affiliations:** 1 Medicine, Taibah University, Medina, SAU; 2 Medicine and Surgery, Taibah University, Medina, SAU; 3 Ophthalmology, Prince Mohammed bin Abdulaziz Hospital, National Guard Health Affairs, Medina, SAU

**Keywords:** implantable collamer lens, keratoconus, nystagmus, refractive surgery, toxic anterior segment syndrome

## Abstract

Toxic anterior segment syndrome (TASS) is a rare but serious postoperative inflammation that can occur after intraocular surgery, including after implantable collamer lens (ICL) implantation. A 27-year-old man with keratoconus and horizontal nystagmus developed severe anterior chamber inflammation two days after toric myopic ICL implantation in the right eye. Unlike endophthalmitis, which presents later with severe pain, conjunctival injection, and vitreous inflammation and requires intravitreal antibiotics, our patient had a rapid onset, no pain, no vitreous involvement, and a quick response to steroids, supporting a diagnosis of TASS. Intensive medical therapy led to full recovery, and subsequent left eye surgery was uneventful. This case highlights the importance of distinguishing TASS from infection through careful clinical reasoning and timely management, ensuring safe toric ICL implantation in patients with nystagmus.

## Introduction

Toxic anterior segment syndrome (TASS) is a sterile, acute postoperative inflammation of the anterior segment caused by a toxic insult to intraocular tissues, such as residues from surgical instruments, medications, or preservatives. Its reported incidence ranges from 0.2% to 0.8% following intraocular surgery [1,2]. Prompt recognition is essential, as management relies on intensive anti-inflammatory therapy to prevent permanent damage to the corneal endothelium and other anterior segment structures. TASS typically develops within 12 to 48 hours after surgery and may closely mimic infectious endophthalmitis, which usually presents later with pain and vitreous involvement and requires intravitreal antibiotics. Phakic implantable collamer lens (ICL) implantation is a well-established option for the correction of high myopia and is generally considered safe. Nevertheless, postoperative complications such as TASS can occur, and reporting these events is important to reflect real-world clinical challenges, facilitate early diagnosis, and guide appropriate management [3]. TASS following ICL implantation in a patient with keratoconus and horizontal nystagmus has been rarely reported. This case report describes the clinical presentation, management, and outcomes in this unique scenario.

## Case presentation

A 27-year-old man with no systemic disease and prior corneal cross-linking (performed six months earlier for keratoconus) was evaluated for refractive surgery. Pre-crosslinking, corneal topography had confirmed bilateral keratoconus associated with a horizontal nystagmus. Preoperative uncorrected distance visual acuity (UDVA) was 0.2 in both eyes, improving to a best-corrected visual acuity (BCVA) of 0.8. Manifest refraction was +0.50/−3.50 × 40 OD and +0.25/−4.75 × 130 OS. Intraocular pressure (IOP) measured 14 mmHg OD and 15 mmHg OS. Anterior chamber depth was 3.7 mm bilaterally, central corneal thickness was 464 µm OD and 463 µm OS, and white-to-white distance was 12.8 mm in both eyes. Endothelial cell counts were 2552 cells/mm² OD and 2493 cells/mm² OS. Endothelial cell counts were measured with the same specular microscope pre- and postoperatively to ensure consistency. Fundus examination of both eyes was unremarkable. Preoperative data are summarized in Table 1.

**Table 1:** Preoperative data of both eyes BCVA: Best-corrected visual acuity; UDVA: uncorrected distance visual acuity; IOP: intraocular pressure

Parameter	Right Eye (OD)	Left Eye (OS)
UDVA	0.2	0.2
BCVA	0.8	0.8
Manifest Refraction	-5.71429	-6.84211
IOP (mmHg)	14	15
Central Corneal Thickness (µm)	464	463
Anterior Chamber Depth (mm)	3.7	3.7
White-to-White (mm)	12.8	12.8
Endothelial Cell Count (cells/mm²)	2552	2493

After thorough counseling regarding the risks and benefits, the patient underwent implantation of a toric myopic ICL (model VTICM5_13.7, sphere -3.0 D, cylinder -4.0 D, axis 135°) in OD under general anesthesia to minimize nystagmus. The surgery was uneventful.

On postoperative day (POD) 1, UDVA in the right eye improved to 0.5, and IOP increased to 30 mmHg; BCVA remained unchanged. Slit lamp examination showed a deep anterior chamber with +1 cells. The transient increase in IOP on POD1 was likely due to the retained ophthalmic viscosurgical device (OVD) or early inflammatory response. It was successfully managed with oral acetazolamide 250 mg twice daily, and IOP normalized by POD2.”

On POD 2, OD demonstrated marked conjunctival injection (+4), anterior chamber reaction of +4 cells with fibrin formation, and a small hypopyon. The IOP had normalized. The patient reported no ocular pain or eyelid edema. B-scan ultrasonography showed no posterior segment involvement. Given the sudden onset of predominant anterior segment inflammation without pain and absence of posterior involvement, TASS was suspected. Empiric intracameral cefazolin, ceftazidime, and dexamethasone were administered.

**Figure 1:**
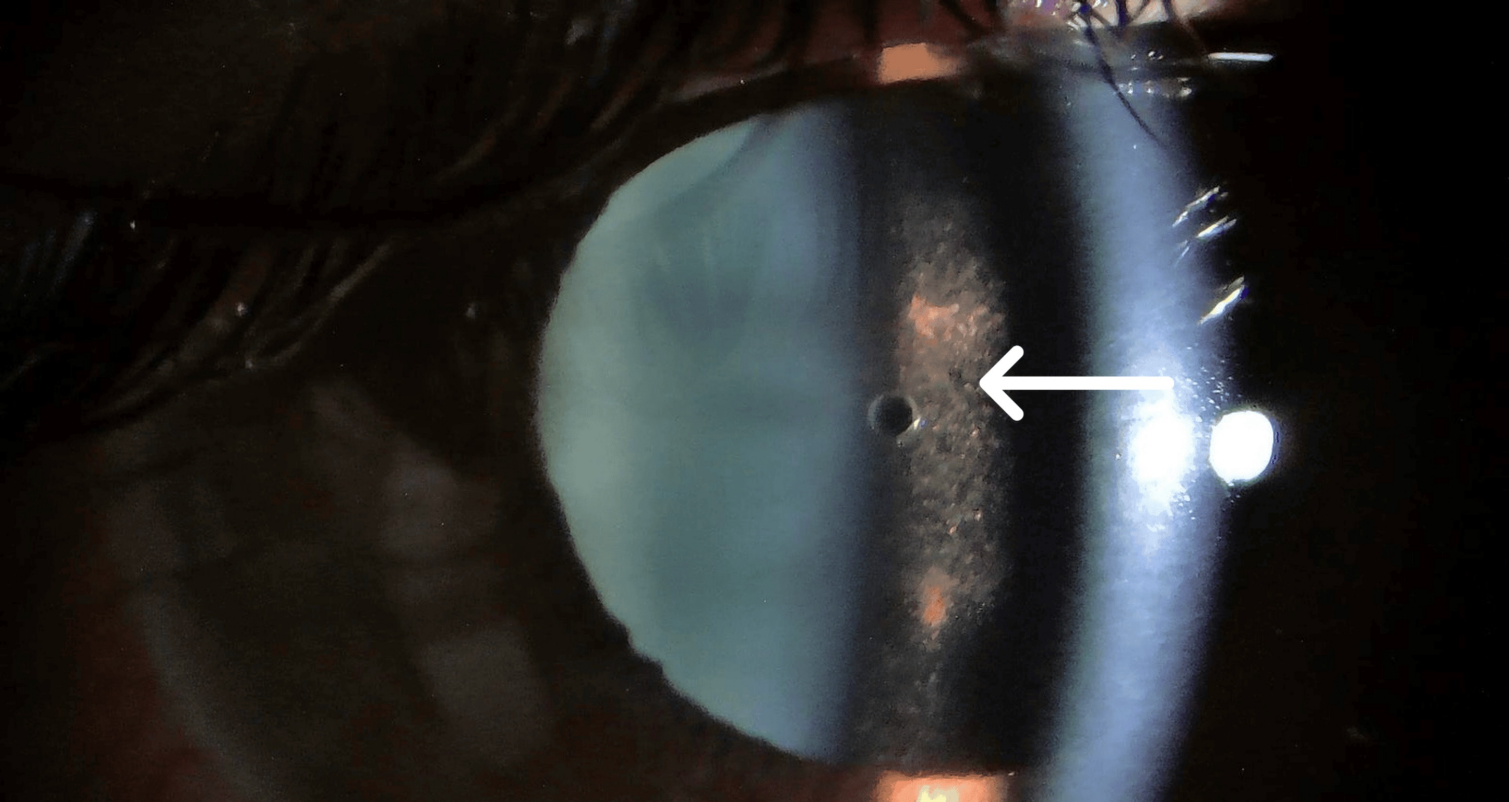
Right-eye exam four days after ICL implantation (TASS) On postoperative day 4 following implantable collamer lens (ICL) implantation, examination of the right eye reveals cells and pigmented deposits in the anterior chamber (indicated by the arrow), consistent with toxic anterior segment syndrome (TASS).

By POD 3, the vision was 0.4 in the right eye, IOP was 13 mmHg, and inflammation had reduced to +2 cells with less fibrin. Anterior chamber inflammation decreased progressively: hypopyon resolved by POD3, fibrin cleared and corneal clarity was restored by POD4, as shown in Figure 1. Serial daily follow-ups showed progressive improvement (Video 1).

**Video 1:** Slit-lamp examination of the right eye post-TASS one month Note the residual effects of toxic anterior segment syndrome (TASS): The video clearly demonstrates persistent iris pigment deposition layered across the implantable collamer lens (ICL) surface, a common sequela of anterior inflammation. Furthermore, the video captures the patient's fine horizontal nystagmus during fixation.

**Figure 2:**
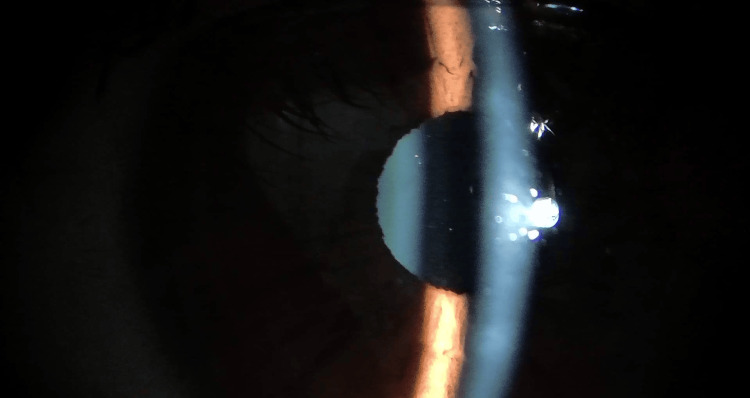
Left-eye exam one week after ICL implantation Figure showing a quiet anterior chamber with no visible cells or pigmented deposits. The cornea and anterior chamber are clear, and the implantable collamer lens (ICL) is well-centered behind the iris, indicating a normal postoperative appearance.

At the follow-up one month postoperatively after full recovery of OD, the patient underwent toric myopic ICL implantation in OS (VTICM5_13.7, sphere -5.50 D, cylinder -4.00 D, axis 26°). That procedure and the postoperative course were unremarkable without any signs of inflammation (Figure 2, Video 2).

**Video 2:** Slit-lamp examination of the left eye one week after ICL implantation This video confirms a quiet anterior chamber and stable position of the implantable collamer lens (ICL) in the left eye one week after surgery. The key observation is the patient's horizontal nystagmus. The video illustrates the positional stability of the ICL within the posterior chamber despite the involuntary, rapid eye movements.

**Figure 3:**
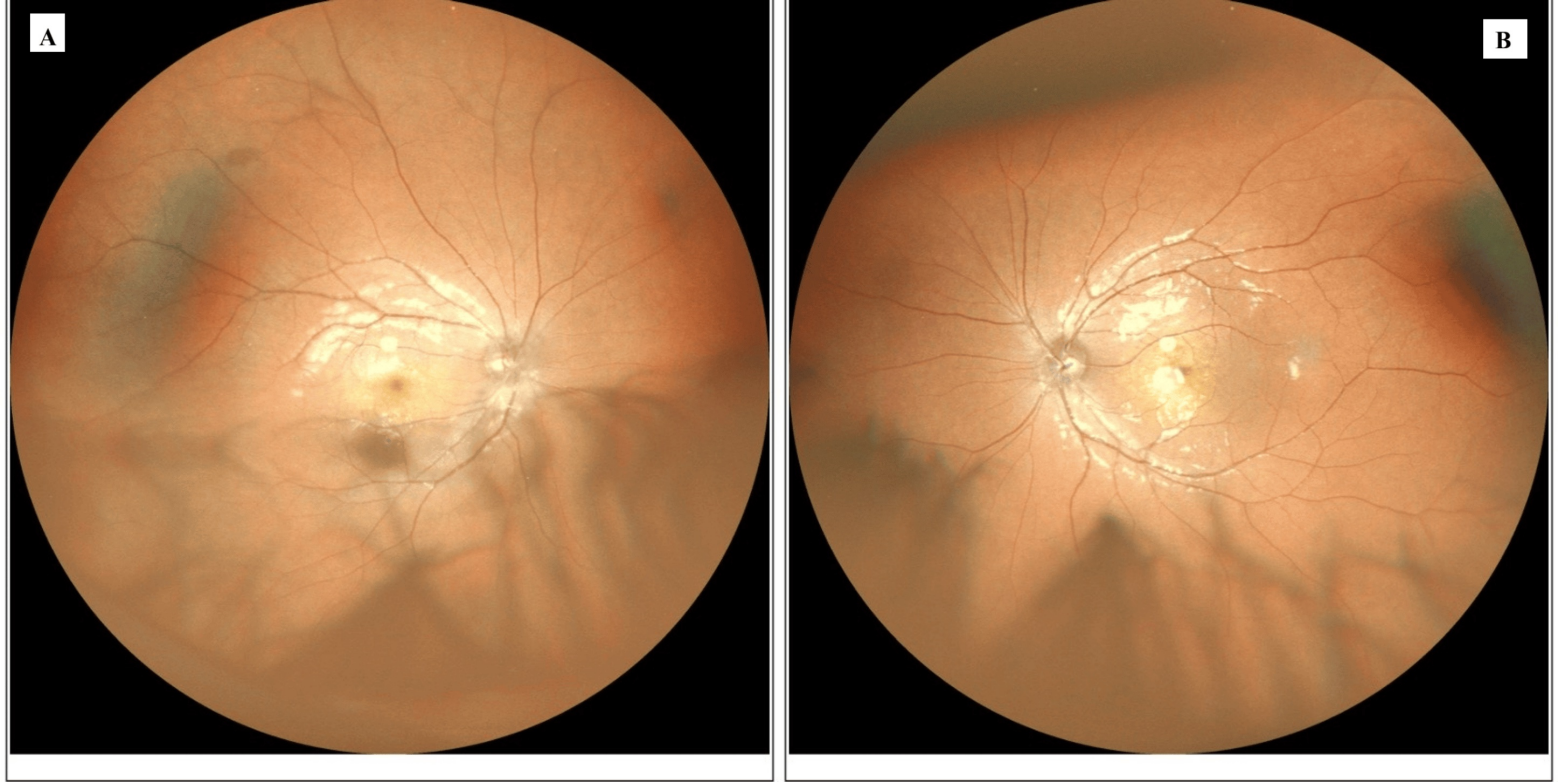
One-month postoperative fundus images of both eyes The posterior segments of both eyes appear stable. The optic discs and maculae are healthy, with no evidence of choroidal or retinal pathology.

At the final follow-up one month postoperatively, the patient’s uncorrected visual acuities were 0.7 OD and 0.9 OS, with clear corneas and well-positioned ICLs in both eyes. The final endothelial cell count was 2517 cells/mm² OD and 2253 cells/mm² OS. Final refraction was near plano (+0.25−0.25×69 OD, +1.00−2.00×159 OS), representing functional success and alignment with preoperative refractive goals (pre-op UDVA 0.2 OD). The fundus photo was unremarkable (Figure 3). The patient was happy and satisfied with his vision. The postoperative clinical course is summarized in Tables 2, 3.

**Table 2:** Postoperative timeline (right eye: TASS) BCVA: Best-corrected visual acuity; UDVA: uncorrected distance visual acuity; IOP: intraocular pressure; TASS: toxic anterior segment syndrome; AC: anterior chamber

POD	UDVA	BCVA	IOP (mmHg)	AC Reaction	Intervention
1	0.5	0.8	30	+1 cells	Oral acetazolamide 250 mg BID
2	0.4	0.8	16	+4 cells, fibrin, small hypopyon	Suspected TASS, topical & systemic steroids, intracameral antibiotics
3	0.4	0.8	13	+2 cells, less fibrin	Continue intensive therapy
4	0.5	0.9	14	Minimal cells	Resolution progressing
30	0.7	0.9	14	Clear	Final outcome

**Table 3:** Postoperative data – left eye BCVA: Best-corrected visual acuity; UDVA: uncorrected distance visual acuity; IOP: intraocular pressure; AC: anterior chamber

POD	UDVA	BCVA	IOP	AC Reaction	Notes
7	0.6	0.8	15	0	Uneventful

## Discussion

TASS is a sterile, acute, postoperative inflammatory reaction affecting the anterior segment of the eye. It typically occurs within 12 to 48 hours after an otherwise uncomplicated intraocular surgical procedure. Patients usually present with decreased visual acuity, ocular discomfort, corneal edema, and significant anterior chamber inflammation, which may include fibrin or hypopyon. IOP may also be elevated. Although TASS is more commonly associated with cataract surgery, it can also occur after phakic intraocular lens implantation, including ICL surgery. While the condition has been rarely reported in the context of ICL surgery, a few recent case series have begun to draw attention to this potential complication. In this case, the patient developed marked anterior segment inflammation with hypopyon within 48 hours following Toric myopic ICL implantation. The hypopyon observed in this case highlights the severity of the inflammation and the importance of early diagnosis and treatment to prevent long-term complications [1,3-5].

The presence of horizontal nystagmus presented challenges for accurate ICL centration and alignment. To control involuntary eye movements, the surgery in OD was performed under general anesthesia, enabling precise axis placement. Special intraoperative measures were taken to stabilize the eye during lens implantation. Postoperatively, the patient demonstrated stable fixation and well-aligned ICL axis, confirming successful management of nystagmus. Furthermore, nystagmus may affect postoperative assessment by limiting the quality of a slit-lamp examination or anterior segment imaging, potentially delaying recognition of complications such as TASS. In this case, the patient’s nystagmus did not interfere with early detection of inflammation, but it remains a consideration in similar clinical scenarios. Additionally, horizontal nystagmus has been associated with altered tear film stability and ocular surface dynamics, which could, in theory, influence postoperative inflammation, although no direct correlation has been established in the literature. This case underscores the need for careful preoperative planning and close postoperative monitoring in patients with nystagmus undergoing intraocular procedures. In addition to the postoperative considerations, the presence of horizontal nystagmus introduced unique challenges in both the preoperative and intraoperative phases. Nystagmus is an involuntary, rhythmic movement of the eyes that may compromise the accuracy of preoperative biometric measurements such as keratometry and WTW distances, which are critical for proper ICL sizing and axis alignment. In our case, particular care was taken during the measurement process to minimize variability and ensure reliable data. Intraoperatively, the patient’s ocular instability posed a potential risk for malalignment of the toric ICL axis or suboptimal centration. However, with deliberate stabilization techniques and meticulous axis marking, the lens was implanted successfully, with no rotational malalignment noted at final follow-up. Although the primary goal of ICL implantation is refractive correction, it is also known that improving visual input in patients with nystagmus, particularly those with a partially sensory origin, can sometimes lead to better fixation stability. However, in our case, and consistent with previous reports (e.g., Bhasin et al., 2021 [6]; Muñoz et al., 2011 [7]), the characteristics of the patient’s horizontal nystagmus, including frequency and amplitude, remained unchanged after surgery. Importantly, despite the dynamic ocular environment, the toric ICL axis remained stable over time, supporting prior findings that well-sized toric ICLs can maintain alignment even in eyes with continuous motion. To our knowledge, there are no prior reports in the literature involving the use of toric ICLs in patients with keratoconus and coexisting nystagmus, making this case a novel contribution to the field [6-9].

A key clinical challenge in postoperative anterior segment inflammation is distinguishing TASS from infectious endophthalmitis, as both conditions can present with anterior chamber cells, hypopyon, and reduced vision. However, several clinical features in our patient supported a diagnosis of TASS. First, the onset of symptoms within 48 hours after surgery aligns with the typical presentation of TASS, whereas endophthalmitis often presents later. Additionally, the absence of ocular pain, corneal infiltrates, and vitreous involvement reduced the likelihood of an infectious etiology. The patient's rapid improvement with intensive corticosteroid therapy further supported a non-infectious inflammatory process. Prompt recognition of these distinguishing features is crucial, as misdiagnosis could lead to unnecessary interventions, including intravitreal antibiotics or surgical procedures. Careful clinical assessment and close postoperative monitoring are essential for accurate diagnosis and appropriate management. Similar to previously reported TASS cases after ICL implantation, the patient exhibited early IOP spike and AC reaction, which resolved rapidly with intensive corticosteroid therapy. No significant endothelial loss or long-term corneal complications were observed, consistent with favorable outcomes [10].

The precise cause of TASS is often difficult to determine, as it is typically multifactorial. Common sources include improperly sterilized surgical instruments, contaminated intraocular fluids, residues from ophthalmic viscosurgical devices (OVDs), manufacturing byproducts on intraocular lenses, and microscopic foreign materials such as lint or fibers. Patient-related factors, such as diabetes, may also predispose individuals to a more severe inflammatory response. In this isolated case, we considered several possible contributors, including retained OVDs, contaminants in the irrigation solution, and endotoxins on surgical instruments. Despite a thorough review of the surgical process, including audits of all single-use instruments, intraocular fluids, and the ICL itself, we were unable to identify a definitive source. The absence of similar cases in other patients and the lack of any detectable breach in protocol limited our ability to trace the exact etiology. This case highlights the complexity of TASS pathogenesis in isolated presentations and underscores the importance of strict intraoperative hygiene and vigilance, even in low-risk procedures [11,12].

## Conclusions

In conclusion, this case highlights a rare but important occurrence of TASS following toric ICL implantation. Prompt differentiation from infectious endophthalmitis and early intensive anti-inflammatory therapy were crucial for a favorable outcome. Additionally, the presence of horizontal nystagmus posed unique preoperative and intraoperative challenges, but with careful planning and surgical technique, toric ICL implantation proved to be a safe and effective refractive solution in this patient. The stable toric ICL axis during follow-up suggests good rotational stability even in dynamic ocular environments. Further long-term studies are needed to evaluate rotational stability, endothelial safety, and visual quality in patients with nystagmus undergoing ICL implantation. Further studies are needed, and strict sterilization protocols may help prevent TASS in future cases.
